# Getting Worse

**Published:** 1857-05

**Authors:** 


### GETTING WORSE.

"The world is worse than it used to was," is the expressed sentiment of many a poor, unfortunate, woe-begone, used-up fellow. His face is as long as a fence-rail—as dolefully serious as Dan Tucker without his dinner—as blue as an indigo-bag. He lives down in the cellar himself, and thinks all the world is doing the same thing. Being of no account, doing nothing, he thinks all creation is like his old shoe, "going down heel," while he is too lazy to pull it up. He is of the *Neverwas* family. Everything and everybody compares unfavorably with the things and bodies of his youth; he excepts himself, of course; and while he is the most striking illustration of going backward, he is a firm believer that he alone of all creation has made progress. Who are the people that will have it that the summers are hotter, the winters colder, the beef tougher, the turkeys smaller, the pigs poorer, the potatoes more watery? they never saw the eggs so small or corn-ears so short; the girls are uglier, the boys ruder; the ministers don't preach as much gospel, nor judges administer the same law; the sun does not shine so bright, nor do the skies look so clear; there is less color in the grass, and less bloom on the rose. In short, the whole world is getting worse, and they are tired of it—in which last the world accords its heartiest reciprocity, for the very good reason, they are of no account to anybody. But who are the persons most given to depreciate the present? Not the money-making man, not the energetic mechanic, who finds he has more than he can do; not the clergyman, whose influences for good pervade a whole community, and whose pulpit is surrounded by respectful multitudes. The fact is, the world is retrograding only to those who are themselves going down hill. When a man begins to croak about "hard times," and about everybody getting worse, the whole world included, it behoves him to inquire if it is not he himself who is thus depreciating in value, in his industry, his activity, his sterling worth, and his high resolution. Energetic men are not croakers. The resolute and those whose motto is "upward"—whose actions show "progress"—are not the men who feel disposed to believe in coming ruin. No; there is progress everywhere—elevation in precept and in practice everywhere around us. In all callings do liberal views prevail. Take the whole question, and let a single fact decide it. Where a dollar was given in private charity a hundred years ago to found a college, endow a seminary, build a hospital, or sustain an asylum, millions are now bestowed. A hundred years ago the pence only were given to humanity; now it is the pound. Be of good courage, then, ye noble workers of good! this world is better for your life, and daily is rising into the more perfect similitude of what it shall be, when, donning its millennial garb, it shall be THE SUN of all worlds!

